# Family Thermometry

**Published:** 1873-08

**Authors:** 


*Family Thermometry; A Manual of Thermometry for Mothers, Nurses, Hospitalers, etc., and all who have charge of the Sick and the Young.* By Edward Seguin, M. D. New York: G. P. Putman & Sons, 1873.

This is an excellent little manual of Thermometry for the use of Mothers and Nurses. We fear however that the mother in cases of sickness can not as a general thing, be prevailed upon to make careful thermometric observations, but will be the rather inclined to rely upon the physician. As a handbook for Nurses and beginners in the study of Human Thermometry it will be of great value, and where heads of families can be prevailed upon to follow its teachings it will doubtless be the medium of much valuable information.

